# Clinicopathological characteristics and survival outcomes of invasive lobular carcinoma in different races

**DOI:** 10.18632/oncotarget.19396

**Published:** 2017-07-19

**Authors:** Li-Yuan Yang, Li-Peng Yang, Biao Zhu

**Affiliations:** ^1^ Department of Intensive Care Unit, Fudan University Shanghai cancer center, Shanghai 200032, China; ^2^ Department of Oncology, Shanghai Medical College, Fudan University, Shanghai 200032, China; ^3^ Department of Pathology, School of Basic Medical Sciences, Fudan University Shanghai 200032, China

**Keywords:** invasive lobular cancer, race, overall survival, breast cancer specific survival, SEER

## Abstract

To investigate the clinicopathological characteristics and to determine whether there is a differential effect of race and examine survival outcomes according to race, 18,295 breast invasive lobular carcinoma (ILC) patients were identified in the Surveillance, Epidemiology, and End Result (SEER) database, which includes White patients (n=15,936), Black patients (n=1,451) and patients of other races (including American Indians/Alaskan Natives and Asian/Pacific Islanders) (n=908). The Black ILC patients presented a higher rate of advanced histological grades and American Joint Committee on Cancer (AJCC) stages, a higher rate of lymph node (LN) involvement and a lower rate of progesterone receptors (PR)-positivity than the White patients and other races. The five-year overall survival (OS) and five-year breast cancer specific survival (BCSS) were worst in the Black patients among these patients (85.5%, 76.0% and 87.7%, P<0.01; 91.1%, 84.4% and 91.6%, P<0.01). Multivariate regression analyses were performed to determine the risk hazards ratios (HR) of death for patients of the White, Black and other races. Among these patients, the Black patients had the worst survival outcomes in five-year OS and BCSS outcomes (HR=1.35, 95% confidence interval (CI) :1.20-1.51, P<0.01; HR=1.39, 95%CI:1.21-1.61, P<0.01, respectively). After a 1:1:1 matching of the three groups, the Black patients still presented worse survival outcomes in BCSS compared to White patients (HR=1.88, 95%CI: 1.14-3.10, P=0.013), however, there was no difference in OS (HR=1.35, 95%CI: 0.93-1.96, P=0.111). Difference in outcomes may partially explained by difference in histological grades, AJCC stage, LN and PR status among the three groups. In conclusion, this study revealed that the Black patients had worse five-year OS and BCSS than White and other race patients.

## INTRODUCTION

Accounting for up to 15% of all breast cancer (BC) cases, ILC represents the second most common invasive histological subtype of BC, after invasive ductal breast cancer (IDC) [1]. It now ranks as the sixth most common cancer in women [2–3]. The traditional concept that ILC arises from the mammary gland lobules and IDC from its ducts is outdated. It is now widely accepted that both ILC and IDC originate from the same micro-anatomical site, namely the terminal duct lobule unit [4]. As the incidence of ILC appears to be increasing, some clinical issues are becoming increasingly important. ILC is pathologically, clinically, and biologically unique among breast cancer tumours. ILC differs from invasive carcinoma in several aspects [5–7]. Clinically, ILC is associated with a higher age at diagnosis, a higher pathological T stage, a higher percentage of multifocal, multicentric and bilateral cases, a higher rate of estrogen receptor (ER) and PR-positivity, a lower histological grade and low tumour cell proliferation [5–10]. Histologically, non-cohesive cancer cells are observed in ILC due to the loss of E-cadherin, either due to mutations inactivating the E-cadherin gene (CDH1), CDH1 haploisufficiency, but this is not observed in other breast cancer subtypes [11]. In addition, the sites of metastasis with ILC are different from those of invasive carcinoma. These patients are indeed associated with more bone and fewer lung metastases, and they tend to develop metastases in unusual sites such as the ovaries, the peritoneum, and gastrointestinal tract [12–14].

Mastectomy is more often required or chosen as a surgical treatment for patients with ILC (approximately 22% to 52%) [15–16]. Moreover, ILC is associated with a higher incidence of positive resection margins after breast-conserving surgery (BCS). Hence, 17% to 65% of ILC patients who undergo BCS require a second surgery for complete resection [17–19]. Most notably, within the first 5 years after diagnosis, ILC tends to have a better disease free and OS than that of IDC [12, 20], but at 6-10 years of follow-up, this trend reversed and a significant advantage was seen for survival in IDC patients.

In the previous study, Garth reported that HR of breast cancer death among ER/PR-positive patients was at least 4 times higher for Black than for White patients [21]. Iqbal and colleagues reported a HR of death from breast cancer of 1.57 for Black compared with White patients [22]. Many researchers have studied ILC, however, a majority of these studies ignored the race of the patients, including White patients, Black patients, American Indians, Alaskan Natives, and Asian and Pacific Islanders. Therefore, we wondered whether there was also some variability of ILC in the different races. By SEER database [23], we aimed to evaluate the epidemiology, histology, staging classification, and survival outcomes of patients diagnosed with ILC on the five-year OS and the five-year BCSS in different races of patients. In addition, we sought to identify the prognostic factors that might account for the survival differences among these races. This study might provide insights into a better understanding of ILC.

## RESULTS

### Clinical characteristics of the study population

Overall, 18,295 patients with ILC were enrolled, including 15,936 White patients, 1,451 Black patients and 908 other patients of other races (including American Indians/Alaskan Natives and Asian/Pacific Islanders). The demographics, tumour characteristics and treatment characteristics of these patients were compared among the races, and the results are summarized in Table 1. There were significant differences in the demographics, including the median age at diagnosis and the marital status. Among the three populations, the White patients were older (60.9±10.9 vs 59.3±11.2 and 57.3±10.9, respectively; P<0.01). In addition, among the three populations, there were considerable differences in the tumour characteristics, including histological grade, AJCC stage, LN status, and PR status. The Black patients presented a higher rate of an advanced histological grade and AJCC stage than those of the White and other races (P<0.01, P<0.01; respectively). Compared to other race patients, the White patients presented higher rate of grade and AJCC stage (P<0.01, P<0.01; respectively). Further more, the rate of LN involvement at diagnosis was higher in the Black patients than White and other races (38% vs 34.8% and 32.8%, respectively; P<0.01). The rate of LN involvement at diagnosis was not different in White than other races (P=0.46). The three populations nearly had the same ER positive rate. PR was expressed in 73.2%, 68.4% and 72.5% of the White, Black and other races, respectively (P<0.01). Expect for what was mentioned above, the treatments were also different among the three populations. The Black patient group had a higher rate of patients who did not receive surgery than the White patient and the other race groups (10.8% vs 5.8% and 6.3%, respectively; P<0.01). Furthermore, the rate of patients who did not receive radiation was higher in the Black patients than that in White and other race (54.4% vs 50.1% and 53%, respectively; P<0.01).

**Table 1:** Patient characteristics in white, black and other race patients^a^

Variance		White	Black	Others	Total	P^b^
		N=15936(%)	N=1451(%)	N=908(%)	N=18295(%)	
**Median age at diagnosis**		60.9±10.9	59.3±11.2	57.3±10.9	60.6±10.9	**<0.01**
**Age at diagnosis**	20-49	2858(17.9)	333(22.9)	256(28.2)	3347(18.8)	**<0.01**
	50-79	13078(82.1)	1118(77.1)	652(71.8)	14848(81.2)	
**Marital status**	Married	9964(60.6)	542(37.4)	601(66.2)	10807(59.1)	**<0.01**
	Unmarried^c^	5654(35.5)	843(58.1)	268(29.5)	6765(37)	
	Unknown	618(3.9)	66(4.5)	39(4.3)	723(4)	
**Laterality**	Right	7808(49)	705(48.6)	444(48.9)	8957(49)	0.957
	Left	8120(51)	745(51.3)	464(51.1)	9329(51)	
	One side	8(0.1)	1(0.1)	0(0)	9(0)	
**Grade**	I	3799(23.8)	296(20.4)	225(24.8)	4320(23.6)	**<0.01**
	II	7274(45.6)	591(40.7)	414(45.6)	8279(45.3)	
	III	1445(9.1)	174(12)	107(6.2)	1726(9.4)	
	IV	72(0.5)	7(0.5)	6(0.7)	85(0.5)	
	Unknown	3346(21)	383(26.4)	156(17.2)	3885(21.2)	
**AJCC stage**	I	6258(39.3)	478(32.9)	347(38.2)	7083(38.7)	**<0.01**
	II	5346(33.5)	489(33.7)	338(37.2)	6173(33.7)	
	III	2754(17.3)	275(19)	123(13.5)	3152(17.2)	
	IV	835(5.2)	134(9.2)	54(5.9)	1023(5.6)	
	Unknown	743(4.7)	75(5.2)	46(5.1)	864(4.7)	
**LN status**	Positive	5551(34.8)	552(38)	298(32.8)	6401(35)	**<0.01**
	Negative	8769(55)	642(44.2)	514(56.6)	9925(54.2)	
	Unknown	1616(10.1)	257(17.7)	96(10.6)	1969(10.8)	
**ER status**	Positive	14263(89.5)	1278(88.1)	813(89.5)	16354(89.4)	0.57
	Negative	655(4.1)	69(4.8)	38(4.2)	762(4.2)	
	Unknown	1018(6.4)	104(7.2)	57(6.3)	1179(6.4)	
**PR status**	Positive	11661(73.2)	993(68.4)	658(72.5)	13312(72.8)	**<0.01**
	Negative	3024(19)	325(22.4)	180(19.8)	3529(19.3)	
	Unknown	1251(7.9)	133(9.2)	70(7.7)	1454(7.9)	
**Surgery type**	Mastectomy	8178(51.3)	693(47.8)	508(55.9)	9379(51.3)	**<0.01**
	Lumpectomy	6771(42.5)	593(40.9)	337(37.1)	7701(42.1)	
	No surgery	918(5.8)	157(10.8)	57(6.3)	1132(6.2)	
	Unknown	69(0.4)	8(0.6)	6(0.7)	83(0.5)	
**Radiation**	Yes	7568(47.5)	622(42.9)	407(44.8)	8597(47)	**<0.01**
	No	7980(50.1)	790(54.4)	481(53)	9251(50.6)	
	Unknown	388(2.4)	39(2.7)	20(2.2)	447(2.4)	

AJCC=American Joint Committee on Cancer, ER=estrogen receptor, PR=progesterone receptor, ILC=invasive lobular carcinoma, LN=lymph node. aIncluding American Indians, Alaskan Natives, Asian and Pacific Islanders and others-unspecified. b*P*-value of the Chi-square test to compare among the three groups. cIncluding divorced, separated, single (never married) and widowed.

### Comparison of the five-year survival among the white, black and other race ILC patients

**Figure 1:**
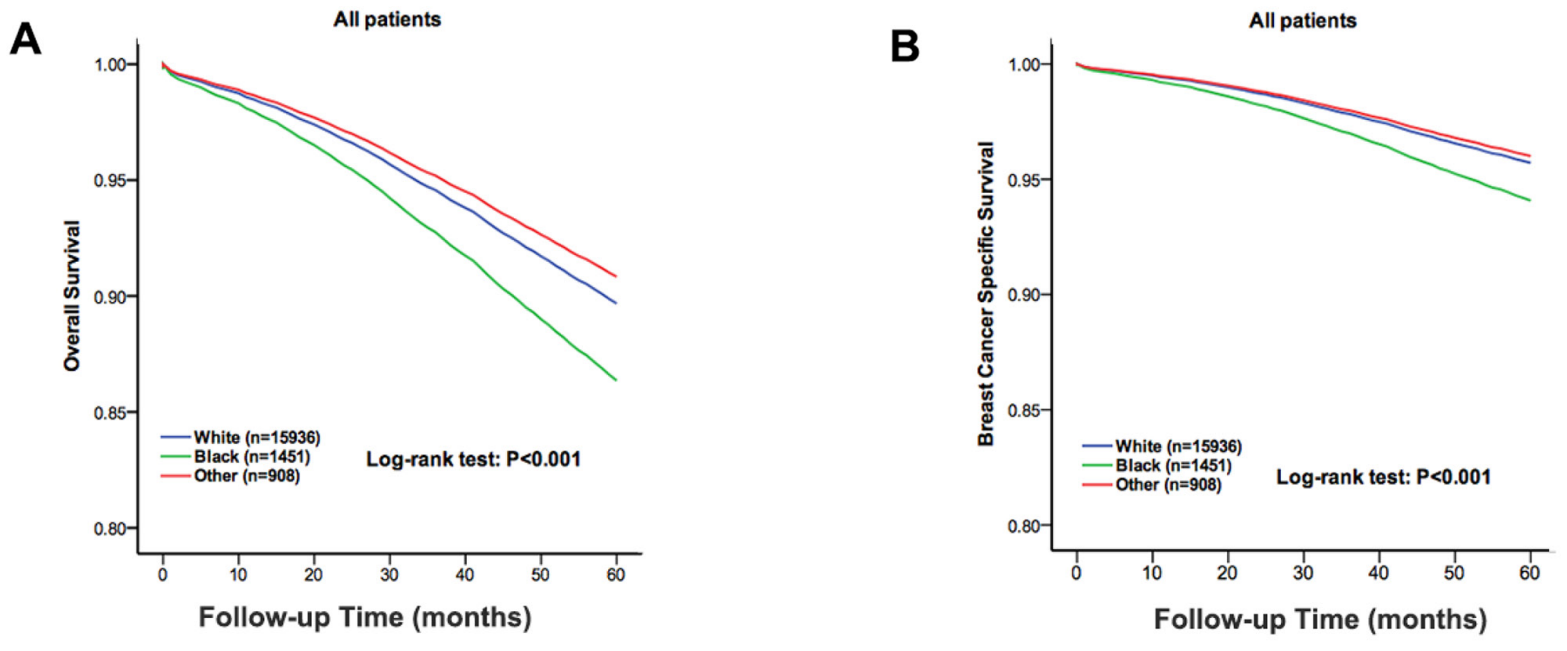
The overall survival and breast cancer specific survival of the white, black and other race patients The Kaplan-Meier test for overall survival (χ^2^ =109.9, *P* < 0.001, **A**) and breast cancer specific survival (χ^2^ =85.3, *P* <0.001, **B**) to compare the White patients to the Black and other race patients.

As shown in the Kaplan-Meier plots, the five-year OS was worse in the Black patients than that in the White and other race patients (χ^2^ =109.9, P<0.01, Figure 1A). The five-year OS rate in the White, Black and other race patients was 85.5%, 76.0% and 87.7%, respectively. We also analysed the five-year BCSS, and a significant difference was observed (χ^2^ =83.5, P<0.01, Figure 1B). The five-year BCSS rate in the White, Black and other race patients was 91.1%, 84.4% and 91.6%, respectively. Furthermore, we used a Cox proportional hazards model to investigate the effects of the clinical characteristics on the five-year OS and BCSS in the multivariate analysis (Table 2). Negative prognostic indicators including old age at diagnosis, an unmarried status, Black race, a high histological grade, a high AJCC stage, LN involvement, a negative ER\PR status and a lack of surgery or radiation were found to be significantly associated with OS and BCSS in the multivariate analysis. When we adjusted the White patients as a control group, race was an independent risk factor for OS in the Black patients compared with the White patients (HR =1.347, 95% CI: 1.201-1.511, *P* <0.01). In contrast, the other races were not an independent risk factors for OS compared with the White patients (HR = 0.883, 95% CI:0.73-1.067, *P* = 0.198). Similar results were observed for the BCSS.

**Table 2:** Multivariate analysis of overall survival (OS) and breast cancer specific survival (BCSS)

Variance		OS		BCSS	
		HR(95% CI)	P	HR(95% CI)	P
**Age at diagnosis**	20-49	Reference	-	Reference	-
	50-79	1.98(1.75-2.23)	**<0.01**	1.47(1.28-1.68)	**<0.01**
**Marital status**	Married	Reference	-	Reference	-
	Unmarried^a^	1.55(1.43-1.67)	**<0.01**	1.37(1.24-1.51)	**<0.01**
**Race**	White	Reference	-	Reference	-
	Black	1.35(1.20-1.51)	**<0.01**	1.39(1.21-1.61)	**<0.01**
	Others^b^	0.88(0.73-1.07)	0.198	0.93(0.74-1.17)	0.538
**Laterality**	Right	Reference	-	Reference	-
	Left	1.01(0.94-1.09)	0.732	1.11(1.01-1.22)	**0.031**
	One side	1.29(0.57-2.91)	0.545	1.26(0.51-3.09)	0.616
**Grade**	I	Reference	-	Reference	-
	II	1.17(1.05-1.31)	**<0.01**	1.32(1.14-1.54)	**<0.01**
	III	1.57(1.37-1.80)	**<0.01**	1.94(1.63-2.31)	**<0.01**
	IV	2.12(1.38-3.26)	**<0.01**	3.68(2.37-5.73)	**<0.01**
**AJCC stage**	I	Reference	-	Reference	-
	II	1.18(1.03-1.35)	**0.021**	1.64(1.30-2.08)	**<0.01**
	III	2.54(2.15-3.00)	**<0.01**	4.58(3.53-5.93)	**<0.01**
	IV	7.23(6.13-8.52)	**<0.01**	16.66(12.95-21.4)	**<0.01**
**LN status**	Positive	Reference	-	Reference	-
	Negative	0.66(0.58-0.75)	**<0.01**	0.44(0.36-0.54)	**<0.01**
**ER status**	Positive	Reference	-	Reference	-
	Negative	1.58(1.37-1.83)	**<0.01**	1.82(1.53-2.15)	**<0.01**
**PR status**	Positive	Reference	-	Reference	-
	Negative	1.50(1.37-1.64)	**<0.01**	1.56(1.39-1.76)	**<0.01**
**Surgery type**	Mastectomy	Reference	-	Reference	-
	Lumpectomy	0.83(0.75-0.92)	**<0.01**	0.72(0.62-0.83)	**<0.01**
	No surgery	1.47(1.27-1.71)	**<0.01**	1.45(1.21-1.73)	**<0.01**
**Radiation**	Yes	Reference	-	Reference	-
	No	1.40(1.28-1.53)	**<0.01**	1.22(1.09-1.36)	**<0.01**

HR=hazard ratio, CI=confidence interval, ER=estrogen receptor, PR=progesterone receptor, ILC= invasive lobular carcinoma, LN=lymph node. The multivariate analysis included the year of diagnosis, age at diagnosis, race, marital status, laterality, grade, LN/ER/PR status, surgery type and radiation. aIncluding divorced, separated, single (never married) and widowed. bIncluding American Indians, Alaska Natives, Asian and Pacific Islanders and others-unspecified.

### Five-year survival analysis of matched groups

**Figure 2:**
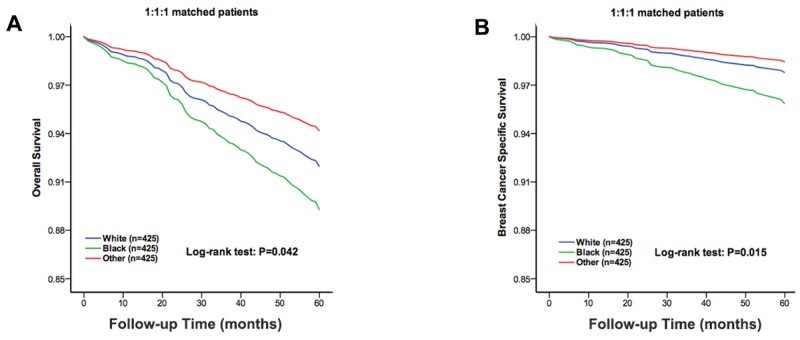
The overall survival and breast cancer specific survival of the 1:1:1 matched groups of the White, Black and other race patients The Kaplan-Meier test for overall survival (χ^2^ =6.361, P=0.042, **A**) and breast cancer specific survival (χ^2^ =8.339, P=0.015, **B**) of the 1:1:1 matched groups to compare the White patients to the Black and other race patients.

There was a big difference among the cases in the three populations. To ensure that the differences in the survival outcomes were not based on baseline differences in demographic and clinical characteristics across races, we performed a 1:1:1 (White: Black: other races) matched case-control analysis using the propensity score-matching method. We obtained a group of 1,275 patients, including 425 patients from each race. For the matched groups, we found no statistically significant difference in the characteristics among the three populations (Table 3). An unadjusted Kaplan-Meier analysis showed the OS and BCSS were worse in the Black patients than those in White and other races (χ^2^ =6.361, P=0.042; χ^2^ =8.339, P=0.015, Figure 2). The OS rate in White, Black and other races was 88%, 84.9% and 90.8%, respectively. The BCSS rate in the White, Black and other race patients was 93.9%, 90.4% and 95.3%, respectively. A Cox proportional hazards model was also used to investigate the effects of the baseline characteristics on OS and BCSS in the multivariate analysis (Table 4). Negative prognostic indicators including old age at diagnosis, an unmarried status, a high AJCC stage and a lack of radiation treatment were found to be significantly associated with OS in the multivariate analysis. However, negative prognostic indicators of BCSS included Black race, an advanced grade, and a high AJCC stage. Race was still an independent risk factor in Black patients compared to that in White patients for BCSS (HR=1.882, 95% CI: 1.141-3.104, P=0.013), but was no longer a risk factor for OS (HR=1.352, 95% CI: 0.933-1.959, P=0.111).

**Table 3:** Patient characteristics in matched groups

Variance		White	Black	Others^a^	Total	P^b^
		N=425(%)	N=425(%)	N=425(%)	N=1275(%)	
**Age at diagnosis**	20-49	82(19.3)	82(19.3)	82(19.3)	246(19.3)	1.000
	50-79	343(80.7)	343(80.7)	343(80.7)	1029(80.7)	
**Marital status**	Married	245(57.6)	244(57.4)	244(57.4)	733(57.5)	1.000
	Unmarried^c^	176(41.4)	177(41.6)	177(41.6)	530(41.6)	
	Unknown	4(0.9)	4(0.9)	4(0.9)	12(0.9)	
**Laterality**	Right	197(46.4)	193(45.4)	193(45.4)	583(45.7)	0.951
	Left	228(53.6)	232(54.6)	232(54.6)	692(54.3)	
**Grade**	I	108(25.4)	108(25.4)	108(25.4)	324(25.4)	1.000
	II	221(52)	217(51.1)	217(51.1)	655(51.4)	
	III	32(7.5)	34(8)	34(8)	100(7.8)	
	Unknown	64(15.1)	66(15.5)	66(15.5)	196(15.4)	
**AJCC stage**	I	193(45.4)	192(45.2)	192(45.2)	577(45.3)	1.000
	II	154(36.2)	154(36.2)	154(36.2)	462(36.2)	
	III	60(14.1)	60(14.1)	60(14.1)	180(14.1)	
	IV	13(3.1)	13(3.1)	13(3.1)	39(3.1)	
	Unknown	5(1.2)	6(1.4)	6(1.4)	17(1.3)	
**LN status**	Positive	146(34.4)	147(34.6)	147(34.6)	440(34.5)	0.997
	Negative	260(61.2)	257(60.5)	257(60.5)	774(60.7)	
	Unknown	19(4.5)	21(4.9)	21(4.9)	61(4.8)	
**ER status**	Positive	415(97.6)	412(96.9)	412(96.9)	1239(97.2)	0.943
	Negative	4(0.9)	4(0.9)	4(0.9)	12(0.9)	
	Unknown	6(1.4)	9(2.1)	9(2.1)	24(1.9)	
**PR status**	Positive	364(85.6)	363(85.4)	363(85.4)	1090(85.5)	0.938
	Negative	55(12.9)	53(12.5)	53(12.5)	161(12.6)	
	Unknown	6(1.4)	9(2.1)	9(2.1)	24(1.9)	
**Surgery type**	Mastectomy	231(54.4)	230(54.1)	230(54.1)	691(54.2)	1.000
	Lumpectomy	178(41.9)	178(41.9)	178(41.9)	534(41.9)	
	No surgery	16(3.8)	17(4.0)	17(4.0)	50(3.9)	
**Radiation**	Yes	207(48.7)	206(48.5)	206(48.5)	619(48.5)	1.000
	No	215(50.6)	216(50.8)	216(50.8)	647(50.7)	
	Unknown	3(0.7)	3(0.7)	3(0.7)	9(0.7)	

AJCC=American Joint Committee on Cancer, ER=estrogen receptor, PR=progesterone receptor, ILC=invasive lobular carcinoma, LN=lymph node. aIncluding American Indians, Alaskan Natives, Asian and Pacific Islanders and others-unspecified. bP-value of the Chi-square test to compare among the three groups. cIncluding divorced, separated, single (never married) and widowed.

**Table 4:** Multivariate analysis of overall survival (OS) and breast cancer specific survival (BCSS) among Races in Matched Patients

Variance		OS		BCSS	
		HR(95% CI)	P	HR(95% CI)	P
**Age at diagnosis**	20-49	Reference	-	Reference	-
	50-79	2.65(1.42-4.94)	**<0.01**	1.96(0.93-4.17)	0.079
**Marital status**	Married	Reference	-	Reference	-
	Unmarried^a^	1.54(1.10-2.15)	**0.012**	1.39(0.86-2.25)	0.181
**Race**	White	Reference	-	Reference	-
	Black	1.35(0.93-1.96)	0.111	1.88(1.14-3.10)	**0.013**
	Others^b^	0.72(0.42-1.09)	0.119	0.70(0.39-1.26)	0.234
**Laterality**	Right	Reference	-	Reference	-
	Left	1.35(0.96-1.89)	0.081	1.32(0.82-2.11)	0.252
**Grade**	I	Reference	-	Reference	-
	II	1.15(0.72-1.83)	0.569	2.50(1.05-5.98)	**0.039**
	III	1.30(0.70-2.42)	0.409	2.83(1.06-7.55)	**0.037**
**AJCC stage**	I	Reference	-	Reference	-
	II	1.36(0.76-2.44)	0.302	2.67(0.88-8.05)	0.082
	III	3.39(1.53-7.51)	**<0.01**	9.41(2.5-35.22)	**<0.01**
	IV	6.80(1.64-28.24)	**<0.01**	16.48(2.2-121.2)	**<0.01**
**LN status**	Positive	Reference	-	Reference	-
	Negative	0.78(0.43-1.38)	0.387	0.63(0.25-1.57)	0.318
**ER status**	Positive	Reference	-	Reference	-
	Negative	1.35(0.54-3.36)	0.521	1.34(0.42-4.30)	0.625
**PR status**	Positive	Reference	-	Reference	-
	Negative	1.38(0.86-2.19)	0.179	0.94(0.47-1.87)	0.851
**Surgery type**	Mastectomy	Reference	-	Reference	-
	Lumpectomy	0.71(0.40-1.25)	0.239	0.62(0.26-1.49)	0.284
	No surgery	3.93(0.38-40.4)	0.25	2.08(0.16-27.71)	0.578
**Radiation**	Yes	Reference	-	Reference	-
	No	1.78(1.10-2.90)	**0.02**	1.52(0.79-2.91)	0.209

HR=hazard ratio, CI=confidence interval, ER=estrogen receptor, PR=progesterone receptor, LN=lymph node. The multivariate analysis included the year of diagnosis, age at diagnosis, race, marital status, laterality, grade, LN status, ER status, PR status, surgery type and radiation. aIncluding divorced, separated, single (never married) and widowed. bIncluding American Indians, Alaskan Natives, Asian and Pacific Islanders and others-unspecified.

### Stratification analysis with molecular subtype

**Figure 3:**
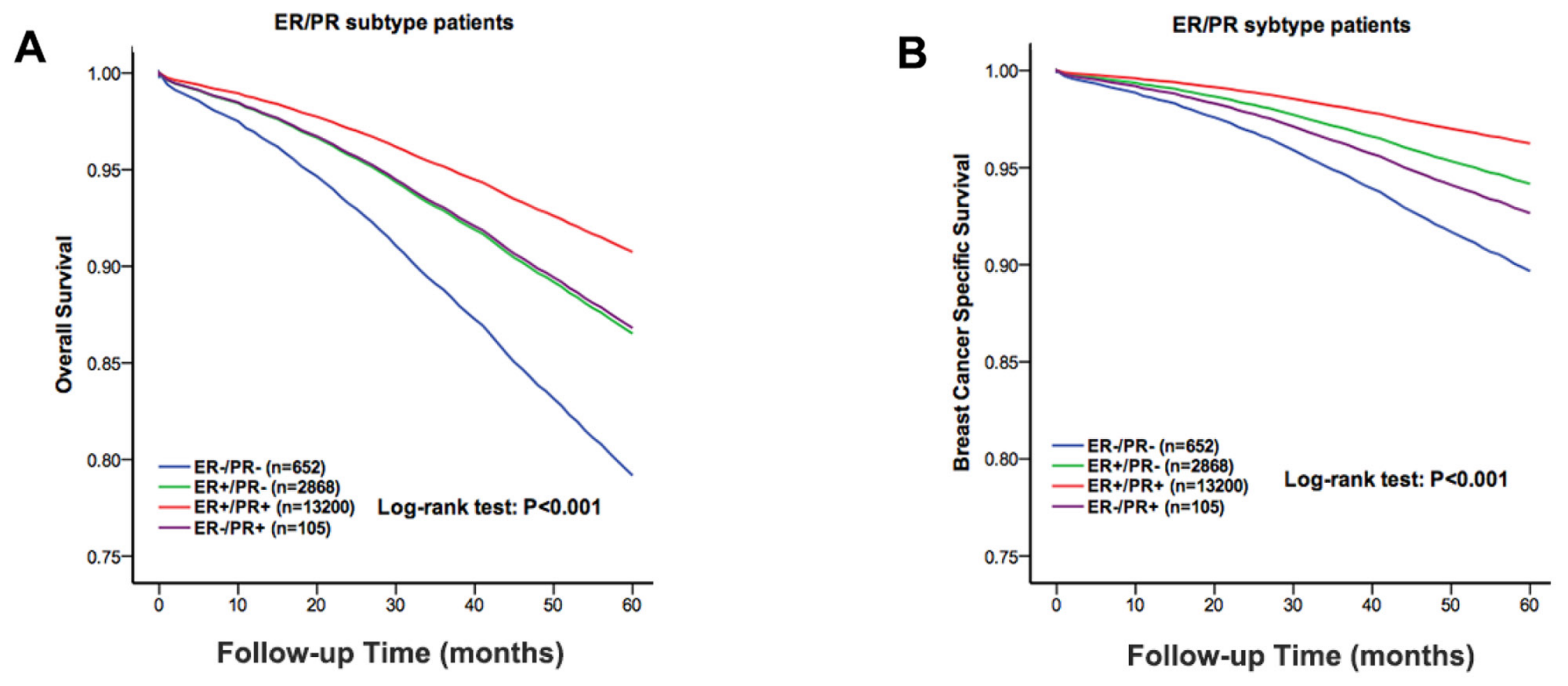
The overall survival and the breast cancer specific survival of the molecular subtype groups of the white, black and other race patients The Kaplan-Meier test for overall survival (χ^2^=103.7, P<0.001, **A**) and breast cancer specific survival (χ^2^=79.4, P<0.001, **B**) to compare the White patients to the Black and other race patients.

To further investigate the effects of molecular subtypes on the ILC outcomes among the different racial patients, we stratified all the cases according to their ER and PR status. Among 16,825 cases, there were 652 ER^-^/PR^-^, 2,868 ER^+^/PR^-^, 13,200 ER^+^/PR^+^ and 105 ER^-^/PR^+^. The subgroup distribution among the White, Black and other race patients was significantly different (P=0.035) (Supplementary Table 1). We further performed a multivariate analysis, stratifying according to molecular subtype (Supplementary Table 2). The Kaplan-Meier analysis showed that the ER^-^/PR^-^ subtype had the worst five-year OS and BCSS among the four subtypes (χ^2^=103.7, P<0.01, Figure 3A; χ^2^=79.4, P<0.01, Figure 3B).

### Subgroup analyses

**Figure 4:**
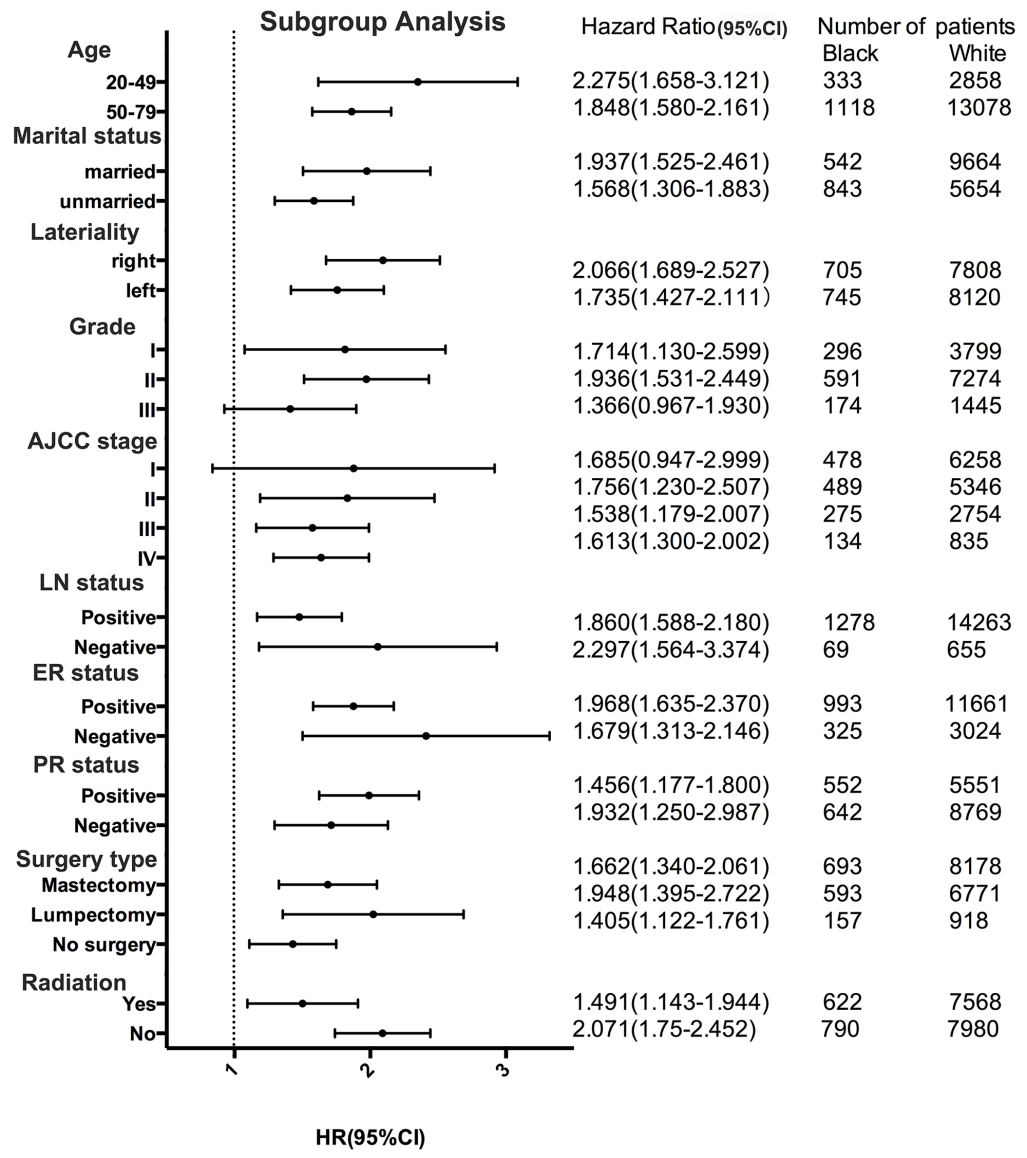
Forest plot of hazard ratios (HRs) for black and white patients with invasive lobular carcinoma (ILC) in the subgroup analysis The diamond on the X-axis indicates the HR and the 95% confident interval (CI) of each subgroup.

A forest plot of HRs that was used to illustrate the exploratory subgroup analyses suggested that in some subgroups Black race was no longer a negative prognostic indicator for BCSS (Figure 4). HR in tumour grade III and AJCC stageIsubgroups were not different between Black and White (HR=1.366, 95%CI: 0.967-1.93, P=0.077; HR=1.685, 95%CI: 0.947-2.999, P=0.076). These results suggest that tumor grade and AJCC stage may be principal confounders in race prognoses.

## DISCUSSION

ILC represents the second most common invasive histological subtype of BC, after IDC [1]. In the Western world, ILC accounts for 10-15% of all breast cancer cases [24–26]. There are a number of the published reports to date that reported ILC. However, there are no research studies examining the ILC in different races, which are necessary to estimate the risk factors unique to different races and furthermore to prevent ILC and optimise therapeutic approaches for treatment in different races. Garth reported that HR of breast cancer death among ER/PR-positive patients was at least 4 times higher for Black than for White patients [21]. Iqbal and colleagues reported a HR of death from breast cancer of 1.57 for Black compared with White patients [22]. In our study, we retrospectively investigated the clinicopathological characteristics and survival outcomes of ILC in different races based on a large population.

Several risk factors of breast cancer preferentially promote ILC development. For instance, late age at first birth, menopausal hormone replacement therapy or late age at menopause confer a higher risk for ILC [27–29]. Several studies have shown racial differences in the use of adjuvant treatment, which could explain observed differences in OS and BCSS among the three groups [30]. Our findings indicate that there were significant differences among the White, Black and other races. For instance, the White patients had a higher median age at diagnosis, a higher rate of PR positivity and a higher rate of radiation than the Black and other races. The Black patients had a lower marital status, a higher histological grade, a higher AJCC stage, a higher rate of LN involvement, a lower rate of PR positivity, a higher rate of those who did not undergo surgery and a lower rate of radiation than the White patients and the other races. In addition, Pestalozzi BC et al [22] reported that patients with ILC were more often being ER-positive and had a lower histological grade. L. Fortunato et al [5–8, 31–33] reported that ILC was associated with a higher age at diagnosis, a higher pT stage, a higher percentage of multifocal, multicentric and bilateral cases, a lower histological grade, a higher rate of hormone receptor (ER/PR)-positivity and a lower rate of HER2 positivity.

Furthermore, we retrospectively researched the survival outcomes of ILC in the different races, and several findings emerged. First, it was evident that the Black ILC patients had a worse outcome based on the five-year OS and BCSS than the White and other race patients. This dilemma has been observed in patients with breast cancer [34]. The survival disadvantage conferred by the Black was likely multifactorial. Second, after the 1:1:1 matching of the three groups by age, marital status, laterality, histological grade, AJCC stage, ER/PR/LN status, surgery type and radiation, an unadjusted Kaplan-Meier analysis showed the OS and BCSS were still worse in the Black patients than those in White and other races. Although, ILC is the most common special breast cancer subtype, almost no studies observe the clinicopathological characteristics and survival outcomes among the different races. Wasif N [21] et al have reported that, most notably, within the first 5 years after diagnosis, ILC tends to have a better disease free and overall survival than of IDC.

The hormone receptor is a known prognostic factor for OS and BCSS. To further investigate the effects of molecular subtype on ILC and the outcomes among the different races of patients, we stratified all the cases according to their ER and PR status. The Kaplan-Meier analysis showed that the ER^-^/PR^-^ subtype had the worst five-year OS and BCSS among the four subtypes.

Racial difference in five-year OS and BCSS may relate to various factors such as accessibility to socio-economic, medical care, use of medical care, biological features and predisposing genetic factors. Socio-economic may be a factor explaining the identified racial disparities. Except for socio-economic, use of medical care may be a major factor explaining the identified racial disparities. It has been documented that Black patients have a lower level of access to health care compared to White and other races patients [34]. Our study also showed that Black patients have a higher rate of those who did not undergo surgery and a lower rate of radiation than the White patients and the other races. Therefore, they are less likely to have timely diagnosis and treatment, receive standard treatment, complete follow-up surveillance and care [35–36].

Inevitably, our study had several limitations. First, our study was limited by the retrospective nature of the analysis using the SEER database and its associated selection bias and missing data. Second, it is certainly possible that given the difficulty in establishing an accurate diagnosis, some of the patients were misclassified or misdiagnosed. Additionally, the effect of the administration of other adjuvant therapies could not be assessed.

In conclusion, this study explored the clinico-pathological characteristics and survival outcomes in White, Black and other races of patients (including American Indians/Alaskan Natives and Asian/Pacific Islanders) with ILC. The Black patients had a poorer five-year OS and BCSS than the White patients and the other races. Furthermore, the similar results were observed after the patients were 1:1:1 matched. Practitioners should continue to strictly follow evidence-based treatment guidelines, and further validation of these results in a large population may help to clarify this issue. An improved clinical and biological understanding of ILC among the three groups might lead to more individualised and tailored therapy for different races of breast cancer patients.

## MATERIALS AND METHODS

### Ethics statement

Our study was approved by an independent ethics committee/institutional review board at the Fudan University Shanghai Cancer Center (Shanghai Cancer Center Ethics Committee). We obtained the SEER research data using the reference number 10581-Nov2015. The data in the SEER database do not require informed patient consent because cancer is a disease reported by every state in the United States.

### Data acquisition and patient selection

We used SEER data released in April 2016, which includes data from 18 population-based registries (1973-2013). Data for the tumour location and histology were recorded according to the International Classification of Diseases for Oncology Version 3 (ICD-O-3). The inclusion criteria used to identify the eligible patients were the following: patients between 20- and 79-years-old, unilateral breast cancer, breast cancer (ICD-O-3 site code C50) as the first and only cancer diagnosis, diagnosis not obtained from a death certificate or autopsy, only one primary site, pathological confirmation of invasive lobular carcinoma not other specified (IDC-NOS) (ICD-O-3 8520/3) with invasion (behaviour code ICD-O-3 malignant), surgical treatment with either mastectomy, lumpectomy or no surgery, and time of diagnosis from 2004 to 2008.

The collected demographic statistics included the age at diagnosis, race and marital status. We treated age at diagnosis as a binary variable that was classified using the following age groups: 20 to 49 years old and 50 to 79 years old. The tumour characteristics included laterality, histologic grade, regional LN status, AJCC stage, ER status, and PR status. The tumour therapy included surgery type and radiation.

### Statistical analysis

The clinicopathological characteristics were compared between the different race groups using Pearson’s Chi-square tests. The Kaplan-Meier method was performed to generate the five-year OS curves and the BCSS curves, and a log-rank test was performed to compare the differences between the curves. Adjusted HRs with 95% CIs were calculated using Cox proportional hazard regression models in order to estimate the prognostic factors.

To account for the differences in the baseline characteristics across the groups, we matched the White, Black and other race patients to 1:1:1 using the following predetermined factors: age at diagnosis, marital status, laterality, histological grade, AJCC stage, LN status, ER status, PR status, surgery type and radiation. We used psmatching3 in SPSS, which was designed for the propensity score matching method and to test the matching quality to determine the balance after the match.

All of the statistical analyses were performed using SPSS statistical software, version 22.0 (IBM Crop, Armonk, NY). A two-tailed P<0.05 was considered statistically significant.

## SUPPLEMENTARY MATERIALS TABLES


