# Supplementary material for: Clinicopathological characteristics and survival outcomes of invasive lobular carcinoma in different races
Source: Oncotarget. 2017 Jul 19;8(43):74287–98. doi: 10.18632/oncotarget.19396 (PMC5650340; doi:10.18632/oncotarget.19396)
Supplement: Supplementary file 1 [file oncotarget-08-74287-s001.pdf]

Clinicopathological characteristics and survival outcomes of invasive lobular carcinoma in different races

SUPPLEMENTARY MATERIALS

Supplementary Table 1: Characteristics of patients with different ER/PR status

Subtype	White	Black	Others[a]	Total	P
	N=14672(%)	N=1316(%)	N=837(%)	N=16825(%)	
ER^-/PR^-	554(3.8)	63(4.8)	35(4.2)	652(3.9)	**0.035**
ER^+/PR^-	2464(16.8)	260(19.8)	144(17.2)	2868(17)	
ER^+/PR^+	11558(78.8)	987(75)	655(78.3)	13200(78.5)	
ER^-/PR^+	96(0.7)	6(0.5)	3(0.4)	105(0.6)	

ER = estrogen receptor, PR= progesterone receptor. P-value was calculated among all groups by the Chi-square test.
[a]Including American Indians, Alaskan Natives, Asian and Pacific Islanders and others-unspecified.

Supplementary Table 2: Multivariate analysis of overall survival (OS) and breast cancer specific survival (BCSS) among ER/PR subtypes

Variance		OS		BCSS	
		HR(95% CI)	P	HR(95% CI)	P
**Age at diagnosis**	20-49	Reference	-	Reference	-
	50-79	1.945(1.713-2.209)	**<0.01**	1.431(1.242-1.649)	**<0.01**
**Marital status**	Married	Reference	-	Reference	-
	Unmarried[a]	1.545(1.424-1.676)	**<0.01**	1.359(1.225-1.508)	**<0.01**
**Race**	White	Reference	-	Reference	-
	Black	1.344(1.19-1.519)	**<0.01**	1.388(1.193-1.616)	**<0.01**
	Others[b]	0.844(0.69-1.033)	**0.1**	0.844(0.656-1.086)	**0.188**
**Laterality**	Right	Reference	-	Reference	-
	Left	1.03(0.951-1.115)	**0.467**	1.147(1.037-1.269)	**<0.01**
	One side	3.748(1.394-10.1)	**<0.01**	4.501(1.668-12.15)	**<0.01**
**Grade**	Ⅰ	Reference	-	Reference	-
	Ⅱ	1.195(1.066-1.339)	**<0.01**	1.395(1.188-1.638)	**<0.01**
	Ⅲ	1.567(1.359-1.808)	**<0.01**	2.044(1.698-2.461)	**<0.01**
	Ⅳ	2.051(1.276-3.295)	**<0.01**	3.722(2.287-6.057)	**<0.01**
**AJCC stage**	Ⅰ	Reference	-	Reference	-
	Ⅱ	1.148(0.993-1.328)	**0.062**	1.613(1.253-2.075)	**<0.01**
	Ⅲ	2.509(2.103-2.994)	**<0.01**	4.562(3.463-6.01)	**<0.01**
	Ⅳ	6.929(5.804-8.271)	**<0.01**	16.14(12.31-21.2)	**<0.01**
**LN status**	Positive	Reference	-	Reference	-
	Negative	0.656(0.57-0.754)	**<0.01**	0.44(0.357-0.544)	**<0.01**
**Surgery type**	Mastectomy	Reference	-	Reference	-
	Lumpectomy	0.834(0.748-0.929)	**<0.01**	0.724(0.624-0.841)	**<0.01**
	No surgery	1.525(1.297-1.793)	**<0.01**	1.538(1.267-1.867)	**<0.01**
**Radiation**	Yes	Reference	-	Reference	-
	No	1.426(1.299-1.565)	**<0.01**	1.243(1.107-1.396)	**<0.01**
**ER/PR status**	ER^-/PR^-	Reference		Reference	
	ER^+/PR^-	0.62(0.532-0.723)	**<0.01**	0.551(0.46-0.66)	**<0.01**
	ER^+/PR^+	0.416(0.362-0.478)	**<0.01**	0.351(0.298-0.412)	**<0.01**
	ER^-/PR^+	0.606(0.379-0.968)	**0.036**	0.7(0.425-1.152)	**0.161**

HR=hazard ratio, CI=confidence interval, ER=estrogen receptor, PR=progesterone receptor, LN=lymph node. The multivariate analysis included the year of diagnosis, age at diagnosis, race, marital status, laterality, grade, LN/ER/PR status, surgery type and radiation. [a]Including divorced, separated, single (never married) and widowed. [b]Including American Indians, Alaskan Natives, Asian and Pacific Islanders and others-unspecified.
